# Heart Disease

**Published:** 1878-12

**Authors:** 


### *HEART DISEASE.*

Of those who die suddenly of supposed "heart disease," not one in twenty has any disease of that organ, as examination after death would conclusively prove. A great majority of these deaths are caused by sudden congestion either of the brain or lungs. There are thousands of heart-sound people who are dragging out miserable lives in constant fear of death from this trouble. They are usually so fixed in their opinions that if an attempt is made to reason them out of the idea, they suspect you are deceiving them, or that you do not understand their case. When the physician once gets the confidence of one of these hypochondriacs, the cure is sometimes astonishingly rapid.

Four months since I was called to see a man who—his physician said—was suffering from hypertrophy, or enlargement of the heart. He is by trade a house painter, but had done no work during the previous six months, and for four weeks had not left his room. I saw at a glance that there were no visible signs of heart trouble, and a most searching examination of the chest failed to reveal any. I soon became satisfied that it was a case of dyspepsia, aggravated, doubtless, by lead poisoning. I ventured to assure him that the heart was sound, and that the frequent and violent palpitation, that he had supposed conclusive evidence of heart disease, were due to other causes, which were carefully explained to him. Treatment was directed to the relief of the actual trouble, and within a week the man was attending to his business; and he says, beyond an occasional, but comparatively slight palpitation that sometimes seizes him, he enjoys excellent health.

The indications of treatment in these cases, and in all cases of disease, are to abstain as far as possible from the use of drugs, and employ in their stead exercise and fresh air. Constipation always exists in dyspepsia, and it is made worse by the frequent use of cathartics. It is in these cases that the value of "Nelaton's Suppositories" is manifested. The world contains two classes of people who go to opposite extremes. One class suffers a thousand deaths from diseases that exist only in the imagination; while by far the larger class seems blind to the existence of troubles whose neglect entails suffering which death alone can relieve.

